# *De-novo* transcriptome assembly and analysis of lettuce plants grown under red, blue or white light

**DOI:** 10.1038/s41598-022-26344-2

**Published:** 2022-12-28

**Authors:** Vinod Kumar, Krishnakumar Sugumaran, Amwaj Al-Roumi, Anisha Shajan

**Affiliations:** 1 Biotechnology Program, Environment and Life Sciences Research Center, Kuwait Institute for Scientific Research, Safat, Kuwait; 2 Desert Agriculture and Ecosystems Program, Environment and Life Sciences Research Center, Kuwait Institute for Scientific Research, Safat, Kuwait

**Keywords:** Biotechnology, Computational biology and bioinformatics, Molecular biology

## Abstract

Lettuce (*Lactuca sativa*) is grown in various parts of the world for use as a leafy vegetable. Although the use of light-emitting diode (LED) in controlled plant production systems has been successfully used to enhance nutritional quality and plant growth efficiently, the molecular basis of lettuce’s response to varying light spectra is not studied. Using next-generation sequencing, we have analyzed the transcriptomes of leaf lettuce (*Lactuca sativa* var. ‘New Red Fire’) grown hydroponically in a modular agricultural production system under three different types of LED lighting: red, blue, and white light. Illumina HiSeq sequencing platform was used to generate paired-end sequence reads (58 Gb raw and 54 Gb clean data) of the transcriptome of lettuce leaves exposed to varying light spectra. The de novo assembled final transcriptome contained 74,096 transcripts. Around 53% and 39% of the assembled transcripts matched to the UniProt and RefSeq RNA sequences, respectively. The validation of the differentially expressed transcripts using RT-qPCR showed complete agreement with RNA-Seq data for 27 transcripts. A comparison of the blue versus red light treatments showed the highest number of significantly differentially expressed transcripts. Among the transcripts significantly up-regulated in blue-light-exposed leaves compared to white-light-exposed leaves, ~ 26% were involved in the ‘response to stress’. Among the transcripts significantly upregulated under red light compared to white light, ~ 6% were associated with ‘nucleosome assembly’ and other processes, such as ‘oxidation–reduction process’ and ‘response to water deprivation’ were significantly enriched. Thus, the result from the current study provides deeper insights into differential gene expression patterns and associated functional aspects under varying light qualities.

## Introduction

Lettuce (*Lactuca sativa* L.) is an economically valuable vegetable crop consumed worldwide. The cultivation and use of lettuce dates back to 4500 B.C. as evident from the tomb paintings in Egypt^1^. The present-day lettuce has gone through centuries of domestication and breeding process to reach the firm-head phenotype from a wild loose and leafy form. The cultivated varieties display different colors and shades of yellow-green to dark red. China, India, the United States, and Spain produce a major proportion of lettuce globally. The total production of lettuce in China is greater than that of all other countries combined^2^. Lettuce is a preferred choice for a health-conscious population owing to its nutritional composition and low calories. Furthermore, it is high in dietary fiber, rich in various vitamins (e.g., A, B9, C, E, K, and thiamine), Ca, Fe, K, Mn, Se, beta-carotene, lutein, anthocyanin, and several phenolic compounds^3^.

The overall appearance including the color, shape, size, texture, flavor, and taste are important factors to consumer acceptance and play a crucial role in fetching premium price in the market. Furthermore, the location of the growing region, season, nutrient inputs, agronomic practices, light parameters, and post-harvest practices can affect the phytochemical content and nutritional composition of lettuce. In modern-day agriculture, lettuce is successfully adapted to the hydroponic-based high-throughput production system.

These systems allow the farmer to use less water and manpower to produce food-safe high-quality produce more precisely^4,5^. Production of plants under a controlled environment has many advantages over conventional farm-based agriculture. Stable production of crops with consistent quality and year-round productivity is possible using plant factory systems. The system allows the production of superior-quality produce for a robust supply chain.

Optimization of lighting in a plant production system enables farmers to produce products with desired appearance, nutrient content, with optimal growth and development in a cost-effective manner. This is possible by testing different light regimes to achieve maximum photosynthetic efficiency in plants. Regulated light intensity and light quality can yield optimized growth and development, coloration, plant architecture, chemical composition, etc.^6–8^. Light quality and quantity play a key role in regulating the biochemical pathways and phytochemical composition of plants and plant products. Accumulation of anthocyanin, various carotenes, and other nutrients are mainly governed by light^9–12^. Anthocyanin biosynthesis has been studied extensively in Arabidopsis^13^. Over twenty-nine anthocyanin molecules have been identified from Arabidopsis which are regulated by high light alone or in combination with exposure to low temperature^14,15^. A review by Thoma et al.,^16^ summarizes the effects on the enhancement of selected metabolites, including anthocyanins, carotenoids, and flavonols. Further, Zoratti et al., compiled various studies related to the role of light on the synthesis and accumulation of flavonoids in various fruit-producing plants^11^. The review provides an overview of the currently known mechanisms of light-controlled flavonoid accumulation and genetic pathways involved in the regulation of flavonoid biosynthesis^11^. Although several reports have shown the regulation of phytochemical biosynthesis and its accumulation^16–18^, the underlying molecular mechanisms of light-induced phytochemical biosynthesis are not well understood in lettuce.

Transcriptome sequencing using high-throughput next-generation technology provides an opportunity to unravel the molecular mechanisms underlying various physiological and biological processes in plants. Wu et al., used *de-novo* sequencing of RNA from the leaf tissues to reveal the light-sensitive regulatory network in *Camellia sinensis* cv. *Baijiguan*^19^. Zhan et al., used RNA sequencing to compare the effects of normal and low temperatures on *Ocimum americanum* var. *pilosum*^20^. Transcriptome sequencing has also been used to understand the gene expression changes associated with cold stress in *Magnolia wufengensis*, a plant of ornamental and economic value^21^. Furthermore, using *de-novo* transcriptome assembly, Zhou and Zhu identified genes that control the biosynthesis of secondary metabolites in *Rhododendron molle*, a traditional Chinese medicinal plant^22^. In a recent study, gene expression analysis was performed using RNA-Seq to understand the influence of different light sources on the regulation of transcriptome involved in leaf aging in leaf lettuce^23^.

Plant growth and development, productivity, coloration, architecture, and chemical composition are governed by various molecular mechanisms, which are modulated by several input triggers, including light, nutrients, and the environment. The genetic basis of such changes induced by variation in the quality of light is still poorly explained and the available data in this context especially for the leafy vegetable crops are sparse. To the best of our knowledge, there are only a few published reports on the molecular changes that occur in the leafy vegetable crops grown in a controlled environment system in repose to changes in incident light quality^24^. In this study, we employed next-generation sequencing technology to investigate the changes in gene expression in leaf lettuce grown under blue, red, or white light emitted by LEDs.

## Materials and methods

### Plant materials and treatments

Lettuce seeds *Lactuca sativa* L. cultivar ‘New Red Fire’, was procured from Vesey Seeds, Canada. The experiments were conducted in compliance with the relevant international guidelines. No additional permissions are required to use the above-mentioned seed material as they were procured from a commercial source.

The seeds were sown in cell plug trays (60 × 41 × 5 cm) containing rockwool pellets (Grodan AO cubes Canada). The seedlings were grown for 15 d in a germination chamber (Percival Model GR-36L, USA) with 24 h photoperiod at a temperature of 20 ± 1 °C. The seedlings were transferred to a Modular Agricultural Production System (MAPS) which was custom-built by JGS Ltd. and the University of Guelph in conjunction with Com Dev International Ltd. (Canada) and Intravision Group AS (Norway) and were grown hydroponically for 35 days under conditions of 20 ± 1 °C temperature, 70 ± 10% relative humidity (Philips Humidifier AC2729/90, The Netherlands), and carbon dioxide levels of 500 µmol mol^−1^ (Module SCD30, Sensiron, Switzerland). Modified Hoagland nutrient solution^25^ was used in recycling mode with a pH maintained at 5.5 ± 0.2 and EC 1.5 dS m^−1^. The three treatments included exposure of lettuce to 100% red (Wavelength 630 nm; LUMILEDS, Philips Lumileds Lighting Company, The Netherlands), blue (Wavelength 460 nm; LUMILEDS, Philips Lumileds Lighting Company, The Netherlands), and white light (Wavelength 400–700 nm with peak at 550 nm; LUMILEDS, Philips Lumileds Lighting Company, The Netherlands) produced by LEDs separately in each level of the MAPS. The photosynthetic photon flux density was measured using a photometer (LI-250A, LI-COR Inc, USA) at a distance of 20 cm above the benchtop. The light intensity was adjusted to 250 µmol m^−2^ s^-1^. Light spectral distribution was scanned using a spectroradiometer (RPS-900R, International Light Technologies, USA) at a distance of 20 cm above the tabletop. A light and dark 18:6 photoperiod cycle was used throughout the experiment.

A total of 54 seedlings were planted at each level for each treatment constituting 162 plants in total. The nutrients were supplied from the same tank for all the treatments and all the parameters were kept identical for the treatments except for light quality. Leaf samples were collected for RNA analysis from the lettuce plants on day 32 of the transfer of seedlings to the MAPS. Fully developed leaf samples were collected using a pair of sterilized scissors from 10 independent replicate plants to form one pooled biological replicate. For maintaining the homogeneity of the samples, multiple leaf samples were collected and pooled from each plant to have an unbiased representation of the leaf transcriptome. For each treatment, two such pooled biological replicates were collected constituting six samples for three treatments. The tissue samples were harvested using sterile scissors, wrapped in labeled aluminum foil, and snap-frozen in liquid nitrogen to preserve the biological status of the collected tissues.

### RNA extraction, library construction, and transcriptome sequencing

The pooled leaf tissues were ground in liquid nitrogen in a prechilled sterile mortar and pestle immediately after sample collection. RNA isolation was performed from 100 mg of the frozen leaf powder using the Sigma Spectrum Plant Total RNA isolation kit (STRN50; Sigma-Aldrich, St. Louis, USA). On-column DNAse digestion was performed according to the instructions provided by the manufacturer (DNASE70, On-column DNAse 1 digestion Set. Sigma-Aldrich, St. Louis, USA). The DNAse-treated RNA was eluted in nuclease-free water and stored at − 80 °C until further use.

The RNA samples were sequenced by BGI Tech Solutions (Hong Kong, China). Total RNA concentration and purity was measured using the NanoDrop™ spectrophotometer (Thermo Scientific, USA). The library was prepared for sequencing using the TruSeq RNA Sample Prep Kit v2 (Illumina Inc., USA). 200 ng total RNA was used as a starting material for purification using the oligo-dT beads. mRNA having a poly (A) tail were fragmented using the Elute, Prime, Fragment mix (Illumina Inc., USA). First-strand cDNA was synthesized by First Strand Master Mix, Super Script II reverse transcription kit (Invitrogen, CA, USA) under the following conditions: 25 °C for 10 min, 42 °C for 50 min, 70 °C for 15 min, followed by addition of second strand master mix, for the synthesis of the second strand at 16 °C for 1 h. Subsequently, the fragmented DNA was subjected to repair using the End Repair Mix by incubating the samples at 30 °C for 30 min. The end-repaired cDNA was further purified using Ampure XP Beads (Agencourt, Beckman Coulter, USA). Further, the samples were subjected to A-tailing and the RNA index adapter was added. The end-repaired cDNA was purified with Ampure XP Beads. Several rounds of PCR amplification with PCR Primer Cocktail and PCR Master Mix were performed to enrich the cDNA fragments. Then the PCR products were purified with Ampure XP Beads. The libraries were amplified on cBot to generate the cluster on the flowcell (TruSeq PE Cluster Kit V3–cBot–HS, Illumina, USA). The amplified flowcell was then sequenced to obtain paired-end reads of 150 bp using the Illumina HiSeq 2000 sequencer.

### Quality analysis and trimming of raw data

A total of 58 Gb paired-end sequencing data of 150 bp read length (163.07 million reads) was checked for quality using FastQC v0.11.4 (https://www.bioinformatics.babraham.ac.uk/projects/fastqc/) before and after trimming. The raw data was trimmed and filtered for low-quality reads based on base-quality score and length, using Prinseq-lite v0.20.4^26^. The minimum Phred quality score and read length considered for trimming was 20 (average quality score for each read) and 50 bp, respectively, allowing maximum Ns of 2.

### *De-novo* transcriptome assembly, filtering, and quality assessment

After the stringent quality filtering and trimming of the raw data, 54 Gb of sequencing data (162.73 million reads) was used for *de-novo* transcriptome assembly using Trinity v2.4.0^27^ with default parameters. The assembled transcriptome was quantified using RSEM tool within Trinity, and lowly expressed transcripts having a TPM of < 1 were excluded. Further, identical transcripts having ≥ 99% identity were removed from the raw assembly using CD-HIT^28^.

The quality of the filtered assembly was assessed by aligning the filtered reads back to the filtered assembly using Bowtie2^29^. Homology-based annotation of the filtered assembly was performed using BLASTx^30^ against UniProt/SwissProt^31^ and BLASTN against NCBI-RefSeq [https://www.ncbi.nlm.nih.gov/refseq/] RNA sequences (both the databases were downloaded in June 2017), with an *E*-value threshold of 1e-10 and maximum of one target sequence.

### Quantification and differential analysis of genes and transcripts

Genes and transcripts were quantified using the filtered assembly across different conditions by RSEM package^32^ within Trinity with default parameters. Differential expression analysis was performed at both gene and transcript levels using edgeR package^33^ using RSEM calculated fragment counts. Significantly differentially expressed gene and isoform clusters between conditions were derived using |log2 fold|≥ 1 (absolute fold ≥ 2) and FDR corrected *p*-value of 0.01. Transcripts differentially expressed in both red and blue light compared to the white light-treated samples were considered for heatmap generation. The heatmap was constructed using the TPM values of the corresponding transcripts in ClustVis tool^34^ by Euclidean distance matrix and average linkage method.

### Validation of differentially expressed transcripts using RT-qPCR

A total of 28 significantly differentially expressed transcripts were selected across the comparisons for validation using reverse transcription-quantitative polymerase chain reaction (RT-qPCR). Ten differentially expressed transcripts (5 up- and 5 down-regulated) in each of the red and blue light conditions compared to white light were selected. Another set of 8 transcripts commonly differentially expressed (5 up- and 3 down-regulated) in both blue and red light compared to white light treatment was also selected for validation. Further, two transcripts (TRINITY_DN16902_c3_g1_i2 and TRINITY_DN14773_c0_g2_i1) that showed no change in their expression in both blue and red light conditions compared to the white light were selected as the control. A total of nine PCR experiments were performed across all the samples. The primer details for all the transcripts considered for validation are provided in Supplementary File 1. Reverse transcription was performed using 250 ng of total RNA (iScript Reverse Transcription Supermix, Biorad, USA), followed by quantitative PCR on a Biorad Real-Time System (CFX96, Biorad, USA) using SYBR green real-time PCR mix (Biorad, USA). The PCR mix was incubated at 95 °C for 5 min and amplification was performed using the following cycling parameters: 40 cycles at 95 °C for 20 s, 60 °C for 20 s, and 72 °C for 20 s, and default melt curve setting were followed. The fold change of the transcripts between the test and control conditions was calculated using 2^−△△^CT method^35^.

### Functional analysis of differentially expressed isoforms

Significant differentially expressed isoforms with thresholds of |log2 fold|≥ 1 and FDR corrected *p*-value of 0.01 were used for Gene Ontology and functional annotations. BLASTx^30^ was used for matching the differentially expressed transcripts against UniProt/SwissProt protein database with an *E*-value of 1e-10, and the results were used to obtain Gene Ontology annotations using Blast2GO^36^. The alignments considered were 20 with a word size of 3, while performing BLASTx^30^. Additionally, to gain better insights into the molecular mechanisms associated with light treatment, the best-matched *Arabidopsis thaliana* proteins (to the significantly differentially expressed isoforms) were analyzed for the enriched annotations, using DAVID Bioinformatics Resources 6.8^37^, and annotations and pathways enriched with a *p* < 0.05 were considered significant.

## Results

### RNA isolation, transcriptome sequencing, and *de-novo* assembly

The extracted RNA concentration was in the range of 210–409 ng/µl and the optical density 260/280 absorbance ratio was ranging between 2.06 and 2.12 indicating high quality of the RNA samples. Illumina sequencing produced around 27 million reads per sample, and after stringent quality filtering, 99.8% of the data was retained across different sample groups corresponding to blue, red, and white light treatment (Table 1).

**Table 1:** Summary of raw data after trimming and filtering.

Group name	Sample name	No. of raw reads	No. of filtered reads	% reads retained after filtering
Blue light	LB1	27,739,156	27,688,404	99.82
	LB2	26,699,224	26,642,794	99.79
Red light	LR1	26,809,398	26,750,588	99.78
	LR2	26,936,856	26,878,440	99.78
White light	LN1	27,991,122	27,936,384	99.80
	LN2	26,889,862	26,834,528	99.79
Total		163,065,618	162,731,138	–

LB: blue light; LR: red light; LN: white light.

After stringent filtering, high-quality sequence data from all the samples were used for *de-novo* transcriptome assembly. The raw assembly contained 125,444 transcripts with an N50 value of 1656 bp (Table 2). Assembled transcripts were filtered to remove low-quality transcripts having TPM (Transcripts per Kilobase Million) value of less than 1, and transcripts with 99% identity. A total of 74,096 transcripts having a length ranging from 201 to 13,702 bp (mean: 1072 bp) were obtained in the filtered assembly. The N50 value of the filtered assembly was found to be 1594 bp (Table 2).

**Table 2:** Statistics of the transcriptome assembly.

	Raw assembly	Filtered assembly
Total sequences (count)	125,444	74,096
Total bases (count)	139,840,620	79,406,940
Min sequence length (bp)	201	201
Max sequence length (bp)	14,393	13,702
Average sequence length (bp)	1,114.77	1,071.68
Median sequence length (bp)	869	833
N25 length (bp)	2394	2297
N50 length (bp)	1656	1594
N75 length (bp)	1013	972
N90 length (bp)	539	503
N95 length (bp)	352	329
As (%)	30.28	30.10
Ts (%)	30.19	29.97
Gs (%)	19.94	20.14
Cs (%)	19.59	19.78
(A + T)s %	60.47	60.07
(G + C)s %	39.53	39.93
Ns %	0.00	0.00

### Quality assessment and annotation of filtered assembly

The quality assessment of the filtered assembly was performed by aligning the reads back to the transcriptome assembly and matching the assembled transcripts to the UniProt/SwissProt and RefSeq databases. Approximately, 95% of the filtered reads were aligned to the generated assembly (Table 3), indicating the completeness of the assembly. Furthermore, approximately 53% (39,242) and 39% (28,653) of the assembled transcripts showed similarity with UniProt/SwissProt protein and RefSeq RNA sequences, respectively, with an *E*-value threshold of 1e-10. The complete list of transcript identifiers along with their matching sequence has been provided in Supplementary file 2.

**Table 3:** Number of reads aligned to the assembled transcriptome.

Group name	Sample name	No. of input reads	Reads mapped (%)
Blue light	LB1	27,688,404	94.6
	LB2	26,642,794	94.4
Red light	LR1	26,750,588	95.1
	LR2	26,878,440	94.3
White light	LN1	27,936,384	94.5
	LN2	26,834,528	94.3

### Quantification of genes and transcripts and differential expression analysis

**Figure 1:**
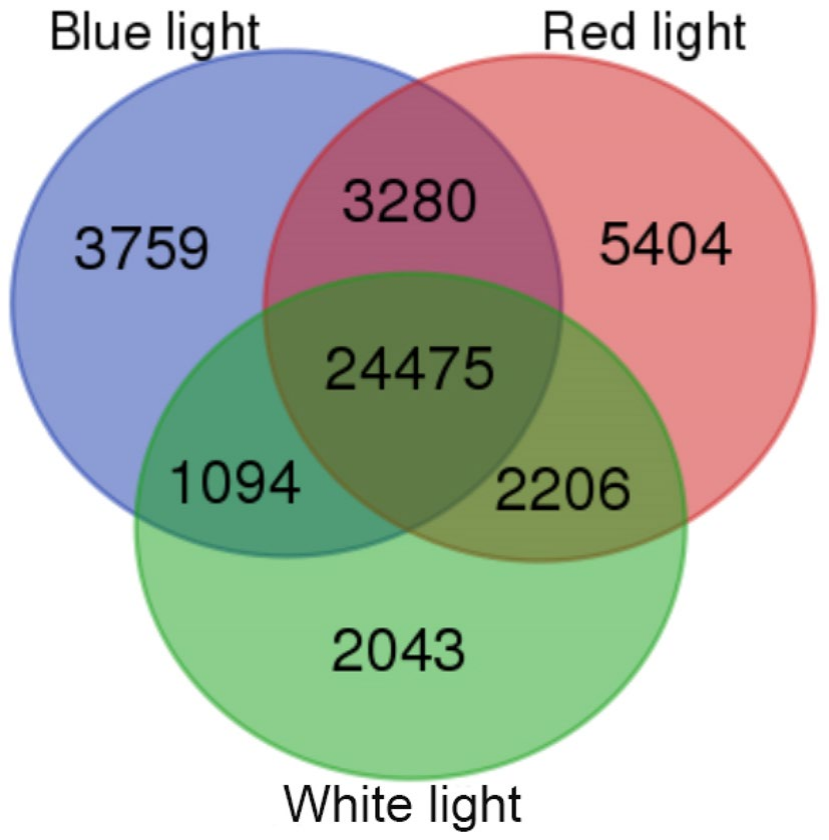
Venn diagram representing the number of common and unique transcripts in lettuce samples grown under blue, red, and white light. Only those transcripts expressed with a minimum TPM value of 1 were considered.

Transcripts with a minimum TPM value of 1 were considered as expressed. Plants grown under red light treatment expressed the highest number while the plants grown under white light expressed the lowest number of transcripts. Furthermore, lettuce grown under the red light resulted in the expression of the highest number of unique transcripts compared to the ones that were grown under blue or white light (Table 4). The number of common and unique transcripts expressed in different samples is shown in Fig. 1.

**Table 4:** Number of genes and transcripts expressed (minimum TPM of 1) in lettuce leaves grown under different light conditions.

Group name	Sample name	No. of genes	No. of transcripts	% of total transcripts	% unique transcripts
Blue light	LB1	26,061	42,371	57.2	8.9
	LB2	25,625	40,956	55.3	8.4
Red light	LR1	27,462	45,374	61.2	10.3
	LR2	27,688	45,949	62.0	11.3
White light	LN1	24,060	38,426	51.9	7.7
	LN2	24,763	39,245	53.0	7.6

**Figure 2:**
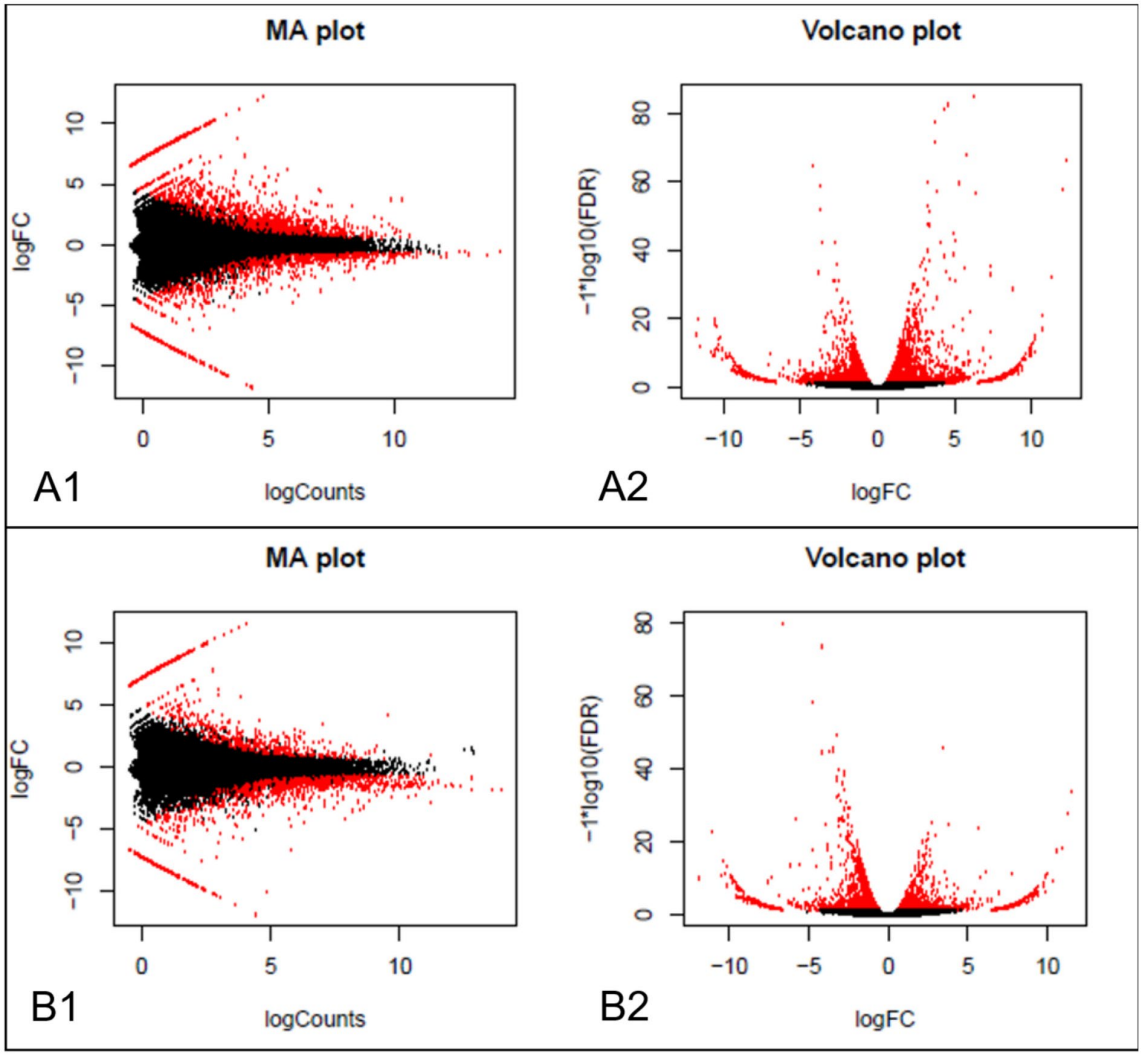
MA and volcano plots representing differentially expressed transcripts. The black dots in the MA plot represent transcripts with similar expression levels, whereas the red dots show significantly up-and down-regulated transcripts. (**A1** & **A2**): Differential expression between blue and white light treatment;(**B1** & **B2**): Differential expression between red and white light treatment; (**A1** & **B1)**. MA plots; (**A2** & **B2**): Volcano plots.

**Figure 3:**
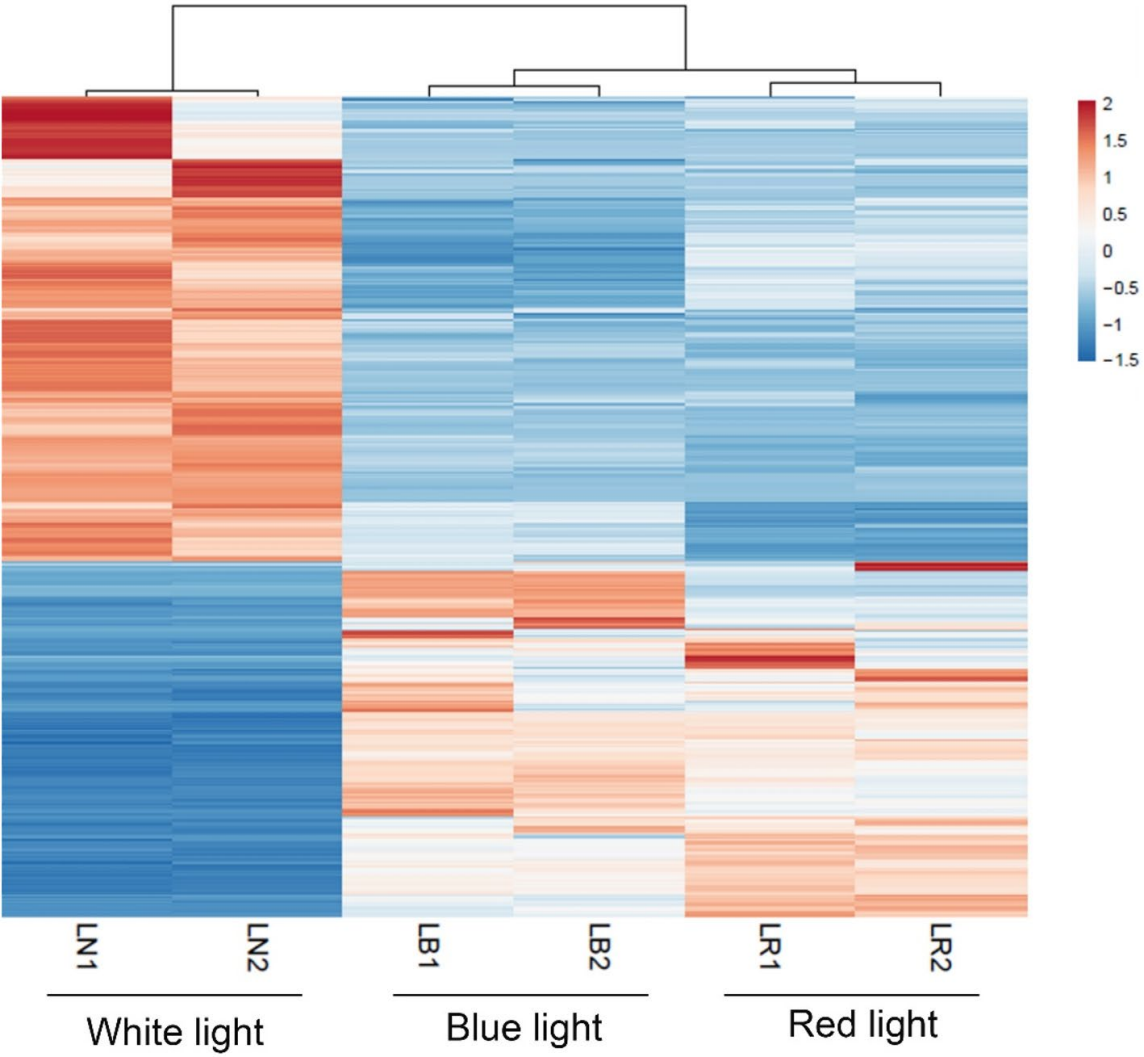
Heatmap representing the expression pattern of transcripts differentially expressed in leaf tissues of plants grown under red light or blue light compared to the ones grown under white light. Hierarchical clustering was performed with the Euclidean distance matrix and average linkage method. Red color indicates higher expression, whereas blue color indicates lower expression of the transcripts. A larger heatmap including transcripts differentially expressed in lettuce plants exposed to red or blue light versus white light is provided in Supplementary Fig. 1. The heatmaps were constructed using the TPM values of the transcripts in ClustVis tool (20th December 2018; https://biit.cs.ut.ee/clustvis/) by Euclidean distance matrix and average linkage method^38^.

A total of 2279 transcripts were found to be significantly differentially expressed between lettuce grown under blue and white light. Among these, 1388 were upregulated, whereas 891 were downregulated in plants grown under blue light compared to white light. A list of top 20 differentially expressed transcripts along with their best-matched SwissProt sequences is provided in Table 5. Furthermore, a total of 1751 transcripts were significantly differentially expressed (upregulated: 547; downregulated: 1204) between lettuce grown under red and white light. Table 6 provides a list of top 20 differentially expressed transcripts and their best-matched SwissProt sequences. Interestingly, the highest number (2745 transcripts) of differentially expressed transcripts were found when leaf RNA samples from plants grown under blue and red light were compared. Among these, 1741 transcripts were upregulated, whereas 1004 transcripts were downregulated in plants grown under blue light compared to red light. A complete list of significantly differentially expressed transcripts across all comparisons can be found in Supplementary file 3. An MA-plot shows log-fold change (M-values) in the log of the ratio of level counts for each gene between the RNA samples from lettuce grown under blue and white light (Fig. 2A) or red and white light (Fig. 2B) against the log-average (A-values, i.e., the average level counts for each gene across the two samples). The heatmap constructed using differentially expressed transcripts in lettuce grown under blue or red or white light treatment showed a clear clustering of the samples of the same groups (Fig. 3).

**Table 5:** Top 20 differentially expressed transcripts between blue and white light treated samples.

Trinity transcript identifier	BLAST matched SwissProt accession (percentage identity)	Gene name/Description	log2 fold	FDR *p*-value
DN18077_c4_g1_i8	Q9M353 (56.4)	CHX20	11.98	2.78E-58
DN17914_c0_g3_i14	F4JKH6 (66.8)	REC2	10.24	3.91E-11
DN15801_c0_g1_i1	I6RE61 (62.4)	Terpene synthase 4	10.16	8.9E-15
DN16823_c1_g4_i1	O49856 (78.2)	FTRC	10.02	1.15E-11
DN18189_c0_g3_i2	Q5XV40 (34.0)	LAZY1	10.01	4.64E-13
DN18094_c2_g2_i1	Q9SRT9 (91.9)	RGP1	9.96	1.05E-12
DN15814_c0_g1_i2	–	–	9.87	6.26E-12
DN17105_c7_g1_i2	–	–	9.66	2.08E-10
DN17609_c1_g3_i4	Q9LIC3 (66.7)	PCMP-H85	9.57	8.17E-09
DN17510_c0_g1_i3	–	–	9.54	1.47E-09
DN17977_c0_g3_i3	Q9SHI1 (75.2)	F20D23.8	−11.79	3.44E-16
DN16848_c0_g3_i7	Q8H118 (71.1)	MLM24.3	−11.58	9.29E-13
DN17208_c0_g5_i1	Q93ZY3 (85.2)	STT3A	−10.79	4.1E-11
DN18192_c3_g7_i8	Q8RY82 (55.4)	T26B15.12	−10.76	4.75E-10
DN16750_c3_g2_i3	O48651 (85.6)	SQE1	−10.65	9.72E-12
DN17360_c4_g1_i1	Q08480 (88.5)	ADK-B	−10.61	2.1E-20
DN16330_c3_g2_i2	Q9FGV1 (64.5)	ARF8	−10.53	2.42E-19
DN12076_c0_g1_i2	–	–	−10.52	2.53E-12
DN17510_c0_g1_i21	–	–	−10.50	2.01E-18
DN17623_c2_g6_i4	–	–	−10.34	6.49E-15

Positive and negative fold changes indicate up- and down-regulation, respectively, in blue light compared to white light exposed samples.

**Table 6:** Top 20 differentially expressed transcripts between red and white light treated samples.

Trinity transcript identifier	BLAST matched SwissProt accession (percentage identity)	Gene name/Description	log2 fold	FDR *p*-value
DN17160_c1_g2_i1	Q38931 (78.3)	FKBP62	11.51	1.64E-34
DN16399_c0_g1_i15	Q8LEV3 (62.4)	T6B20.5/T6B20.4	10.96	5.08E-19
DN16926_c2_g2_i3	P49690 (97.7)	RPL23A	10.29	3.39E-10
DN17692_c0_g1_i5	Q94BX4 (82.6)	PIGA	9.9	5.89E-07
DN18387_c2_g2_i18	F4I5Q6 (47.0)	XI-A	9.43	1.65E-08
DN17965_c1_g1_i13	–	–	9.26	9.59E-08
DN17647_c0_g2_i11	–	–	9.22	2.09E-06
DN16367_c3_g5_i2	–	–	9.12	5.41E-07
DN18111_c3_g2_i14	–	–	9.12	3.81E-06
DN16893_c1_g2_i4	Q9FN11 (53.0)	LBD37	9.12	9.88E-07
DN17027_c2_g4_i3	Q39033 (71.0)	PLC2	−11.91	9.32E-11
DN16948_c2_g1_i10	Q84V03 (31.2)	F16F14.15	−11.06	1.41E-23
DN17343_c1_g9_i1	Q9LP46 (32.0)	SCAR3	−10.51	1.06E-11
DN17642_c0_g1_i13	P45739 (94.0)	Catalase	−10.11	9.22E-09
DN18289_c4_g2_i9	Q8LCQ4 (82.2)	LHCA6	−9.90	1.73E-11
DN18008_c4_g1_i6	Q9SWF9 (79.5)	–	−9.89	1.81E-11
DN17073_c1_g1_i7	Q8GYW8 (32.8)	SCT	−9.82	9.78E-11
DN16344_c1_g1_i4	O65718 (70.7)	CNGC2	−9.73	7.54E-10
DN16335_c0_g4_i4	Q07346 (85.2)	GAD	−9.58	1.73E-05
DN17847_c1_g2_i6	Q9M2N5 (25.3)	HAT	−9.55	6.81E-08

Positive and negative fold changes indicate up- and down-regulation, respectively, in red light compared to white light exposed samples*.*

### Validation of differentially expressed transcripts

A total of 28 significantly differentially expressed transcripts were considered for validation using RT-qPCR. However, one transcript, TRINITY_DN17554_c0_g6_i2 was excluded as it showed multiple bands. All 27 transcripts when tested using RT-qPCR analysis, showed complete agreement with the RNA sequencing data. The transcripts were differentially expressed with a minimum |log2 fold|> 2 according to RT-qPCR (Table 7).

**Table 7:** RT-qPCR validation of *de-novo* assembled differentially expressed transcripts.

*De-novo* assembled transcript ID	Experiment type	Differential expression of transcripts (Log2 fold)	
		RT-qPCR	RNA-Seq
TRINITY_DN17105_c7_g1_i2	LB vs. LN	7.03	9.66
TRINITY_DN17085_c3_g8_i2	LB vs. LN	3.65	3.89
TRINITY_DN17258_c5_g6_i1	LB vs. LN	4.12	4.51
TRINITY_DN14439_c0_g1_i2	LB vs. LN	5.25	5.27
TRINITY_DN16921_c1_g1_i1	LB vs. LN	2.85	2.74
TRINITY_DN17088_c4_g1_i1	LB vs. LN	−5.88	−3.78
TRINITY_DN8339_c0_g1_i2	LB vs. LN	−3.57	−3.53
TRINITY_DN16809_c0_g1_i1	LB vs. LN	−4.68	−4.22
TRINITY_DN16676_c2_g5_i3	LB vs. LN	−3.41	−3.41
TRINITY_DN13939_c0_g2_i1	LB vs. LN	−3.42	−3.37
TRINITY_DN15652_c0_g1_i2	LR vs. LN	6.00	6.29
TRINITY_DN14960_c1_g2_i1	LR vs. LN	3.02	3.12
TRINITY_DN19789_c0_g1_i1	LR vs. LN	3.21	4.04
TRINITY_DN16595_c1_g4_i1	LR vs. LN	2.58	3.43
TRINITY_DN16803_c0_g8_i1	LR vs. LN	2.59	2.87
TRINITY_DN4947_c0_g2_i1	LR vs. LN	−7.56	−5.27
TRINITY_DN22916_c0_g1_i1	LR vs. LN	−3.45	−4.32
TRINITY_DN17330_c0_g3_i1	LR vs. LN	−3.05	−3.21
TRINITY_DN21813_c0_g1_i1	LR vs. LN	−5.00	−8.17
TRINITY_DN26970_c0_g1_i1	LB vs. LN & LR vs. LN	14.44; 9.82	12.26; 7.74
TRINITY_DN11681_c0_g2_i1	LB vs. LN & LR vs. LN	5.38; 4.02	5.76; 3.64
TRINITY_DN3588_c0_g1_i1	LB vs. LN & LR vs. LN	4.88; 2.44	5.06; 2.42
TRINITY_DN12130_c0_g1_i1	LB vs. LN & LR vs. LN	5.44; 5.46	5.92; 4.94
TRINITY_DN11791_c0_g1_i1	LB vs. LN & LR vs. LN	9.64; 7.31	8.79; 5.68
TRINITY_DN16966_c2_g7_i2	LB vs. LN & LR vs. LN	−2.97; -5.10	−2.3; -5.78
TRINITY_DN15099_c0_g3_i2	LB vs. LN & LR vs. LN	−3.82; -6.25	−3.18; -6.15
TRINITY_DN15099_c0_g3_i3	LB vs. LN & LR vs. LN	−3.60; -5.71	−7.48; -7.57

LB: Blue light; LR: Red light; LN: white light.

### Functional analysis of differentially expressed transcripts

**Figure 4:**
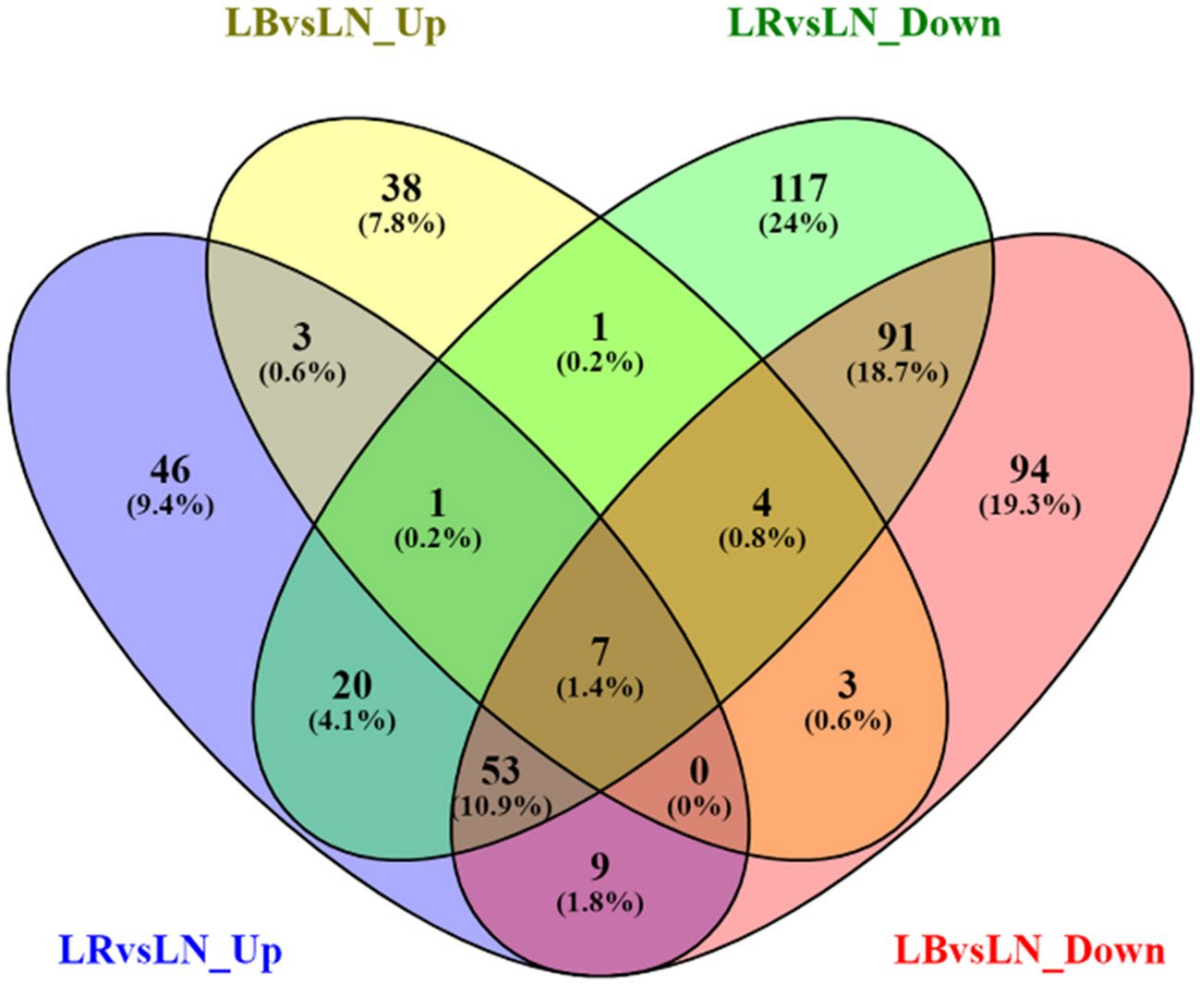
The number of shared and unique biological processes across different light treatments. LB: Blue light; LR: Red light; LN: white light.

**Figure 5:**
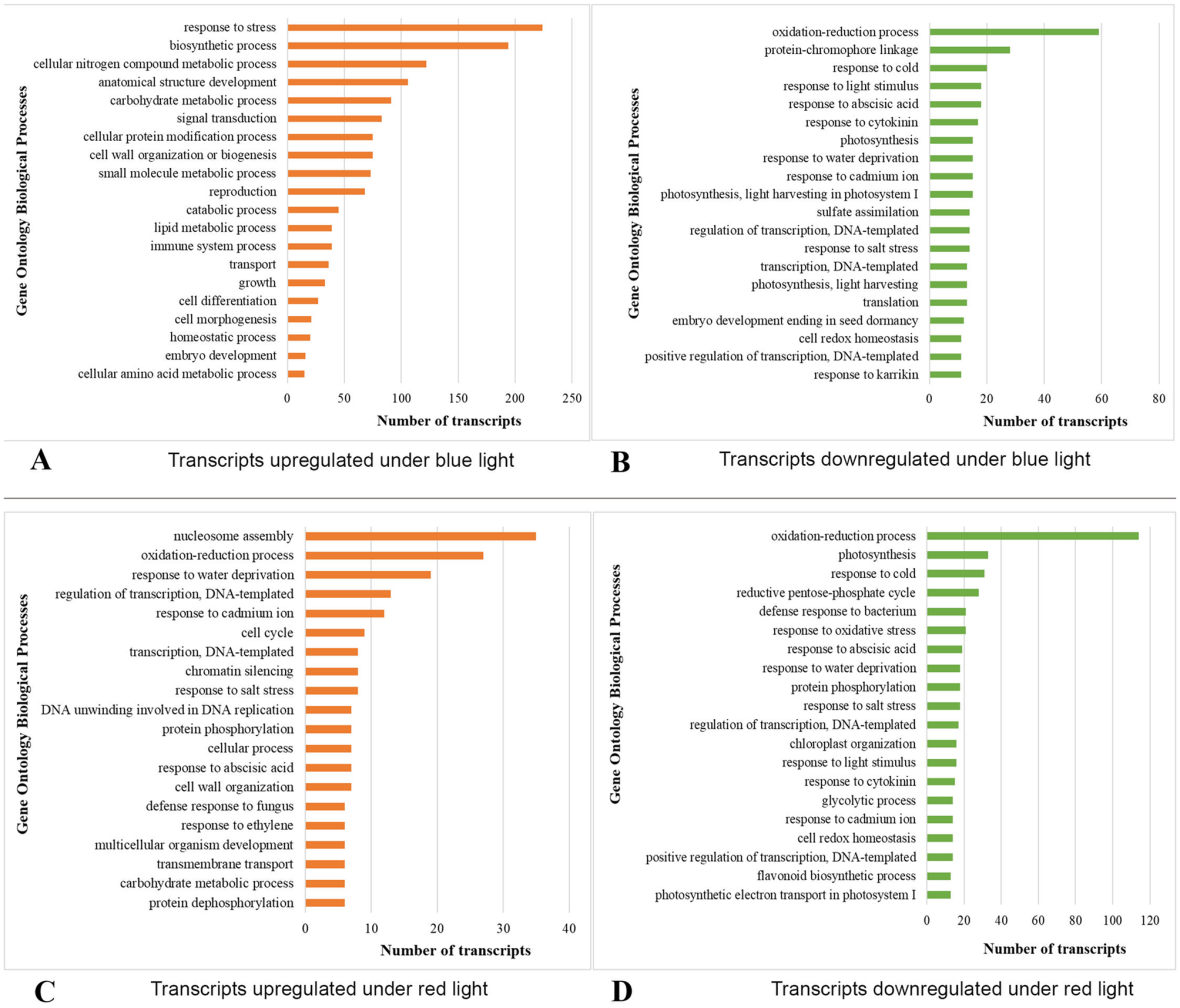
Top 20 Gene Ontology biological processes for differentially expressed transcripts between blue (**A** & **B**) or red (**C** & **D**) light treated lettuce. (**A**) Transcripts upregulated in leaf tissues of lettuce grown under blue light; (**B**) Transcripts downregulated in leaf tissues of lettuce grown under blue light; (**C**) Transcripts upregulated in leaf tissues of lettuce grown under red light; (**D**) Transcripts downregulated in leaf tissues of lettuce grown under red light.

The significantly differentially expressed transcripts were mapped to gene ontology annotations of different species. Our results showed that majority of the annotated transcripts matched UniProt/SwissProt sequences of *Arabidopsis thaliana*. This could be due to the high-quality characterization of *A. thaliana* genome and transcriptome. Furthermore, functional analysis of the significantly differentially expressed transcripts resulted in different sets of annotations in blue vs. white and red vs. white light-exposed lettuce plants. Isoforms downregulated in lettuce plants grown under red light resulted in the most number of unique biological processes followed by that of blue light (Fig. 4). The biological processes, such as photosynthesis, cell cycle, secondary metabolic process, signal transduction, and protein folding were represented by the upregulated unigenes by both blue and red light exposure. Approximately, 26% of the significantly upregulated transcripts in blue vs. white light were found to be involved in ‘response to stress’. Further, the upregulated unigenes were found to be involved in various metabolic and biosynthetic processes, such as cellular nitrogen metabolic processes, carbohydrate metabolism, and lipid metabolism. A few of the transcripts were also found to be involved in biological processes related to transport, cell differentiation and morphogenesis, and homeostasis (Fig. 5A). Furthermore, the downregulated transcripts were found to be involved in various processes (Fig. 5B). The transcripts significantly upregulated in plants grown under red vs. white light were found to be involved in processes such as nucleosome assembly, oxidation–reduction process, cell cycle as well as various DNA replication-related processes (Fig. 5C). Interestingly, many similar biological processes were downregulated by both red and blue light treatment (Fig. 5B and D). A few of these processes include ‘oxidation–reduction’, ‘response to light’, ‘response to cytokinin’, ‘response to water deprivation’ and ‘photosynthesis’. A complete list of GO annotations (with at least two isoforms involved) along with the transcript identifiers is provided in Supplementary file 4.

As the *A. thaliana* genome is well annotated, we performed GO and pathway enrichment analysis using the best matched Arabidopsis proteins to understand the mechanisms underlying the blue or red light exposure. The analysis identified several GO biological processes and KEGG (Kyoto Encyclopedia of Genes and Genomes database^39^) pathways to be significantly enriched in each pair-wise comparison. We found ‘response to water deprivation’ to be enriched significantly both in blue and red-light treatment groups. Interestingly, ‘response to blue-light’ was significantly enriched by the upregulated genes in blue vs white light exposure. The KEGG pathway ‘plant hormone signal transduction’ was enriched by the upregulated genes between blue and white light treatment. The Supplementary file 5 provides all the processes and pathways enriched across each pair-wise comparison using best matched Arabidopsis proteins.

## Discussion

RNA-sequencing of paired-end data corresponding to leaf lettuce grown under blue, red, or white light was generated using Illumina HiSeq sequencing platform. The raw data was filtered for low-quality reads, and a *de-novo* transcriptome assembly was constructed. The low-coverage (TPM < 1) and redundant sequences (99% identity) were filtered out from the raw assembly to obtain the final assembly. The filtered assembly contained a set of 74,096 unigenes with an N50 value of 1594, which was comparable to a recent study published on red leaf lettuce^40^.

Based on the overlapping of transcripts across different light conditions, we found that the overall transcriptome of the lettuce plant appears to be conserved. A total of 24,475 transcripts were shared across the tested treatment sets consisting of RNA isolated from leaf lettuce grown under blue, red, and white light. Furthermore, a total of 5404 transcripts were expressed exclusively in plants grown under red light, whereas 3759 transcripts were exclusively expressed in plants grown under blue light, which indicates that the red light treatment may activate the expression of a large number of isoforms compared to that of blue or white light treatment.

Around 95% of the filtered reads were aligned back to the assembled transcriptome, indicating a high degree of accuracy of the generated assembly. The final filtered assembly was functionally annotated, and approximately 53% of the assembled transcripts matched to the UniProt/SwissProt sequences with high accuracy (E-value of 1e-10).

Differential expression analysis indicated a significant number of genes to be up/down regulated between blue/red and white light treated lettuce samples. Cation/H + exchanger 20 (CHX20) was the most significantly upregulated unigene when lettuce was grown under blue light treatment, whereas translation initiation factor 2, small GTP-binding protein (FUG1) was the most significantly downregulated isoform. CHX20 is a member of putative Na + /H + antiporter family and is involved in osmoregulation and possibly pH modulation. FUG1 is localized in chloroplast and functions similar to translation initiation factor 2. Furthermore, Rotamase FKBP 1 (ROF1), upregulated by red light treatment with the highest fold modulates thermotolerance by interacting with HSP90.1, while phosphoinositide phospholipase C 2 (PLC2), a downregulated gene (with a fold of 11.9) by red light treatment is known to be involved in signal transduction. In an earlier study, Kitazaki et al. investigated the growth, development, and molecular response of lettuce plants under two levels of light intensity (PPFD 100 or 300 µmol m^−2^) and five different light qualities (white fluorescent light, blue 470 nm, green 510 nm, green 520 nm, and red 680 nm)^24^. In this study, although the metabolite analysis was performed at three-time points zero, one, and seven days, the gene expression studies were performed at zero and one-day time points following exposure to different light qualities^24^. Kitazaki et al. documented the changes in transcriptome occurring at the very early stage, (0 and 24 h) in the third leaf of lettuce plants. However, we have investigated the gene expression at a stage when the lettuce is fully developed and ready for harvest. Some of the genes, *CHS, CHI, CHI-like 1, F3H F’3H, DFR,* and *FLS* were upregulated at the 24 h timepoint in blue light exposed plants^24^. However, we have noticed a significant difference in the expression of genes and pathways (Tables 5, 6 and 7, Fig. 5) which were not recorded in the earlier report^24^. The reason for this could be due to differences in the time point chosen for the gene expression study. Also, the early light responsive genes may not maintain the same level of differential regulation even during the late maturation stage of thirty-two days under three different light qualities in a controlled MAPS system.

The morphological and developmental changes in lettuce grown under three different light qualities are documented in an earlier report^41^. The blue light exposed lettuce plants were significantly taller and intense red in color, whereas, red light exposure resulted in green coloured leaves with significantly higher number of leaves per plant compared to the other two counterparts^41^. The accumulation of red color due to the accumulation of anthocyanin in response to exposure to blue light has been reported in *Arabidopsis*^42^, tomato^43^, pear^44,45^, *Brassica napus*^46^*,* lettuce^47^, many plant species^48–52^. A broader time course analysis at various developmental stages of plants following different light exposure would help further understanding of gene regulation linked to pigment accumulation.

The functional annotation analysis of the differentially expressed unigenes indicated 11 biological processes to be common in red and blue light treated leaf samples. A few of these include photosynthesis, secondary metabolic process, and cell cycle. A study by Manivannan et al., showed an increase in the production of secondary metabolites by blue light followed by red light treatment of *Scrophularia kakudensis*, a potential medicinal plant^53^. Furthermore, several biological processes enriched by the differentially expressed unigenes in the current study have been also reported by other studies in response to light stimulation^54^.

To further understand the role of differentially expressed unigenes, we performed functional annotation using Arabidopsis protein identifiers that matched the *de-novo* assembled unigenes. The results showed enrichment of similar GO annotations that were obtained by the functional analysis of differentially expressed unigenes. The biological processes, such as ‘response to blue light’, ‘circadian rhythm’, and ‘photosynthesis’ were significantly enriched by the differentially expressed genes by blue light treatment, which is in agreement with the published studies^53,54^. The stomata are important channels for the exchange of materials, such as gas and water with the external environment^55^. A study by Muneer and co-workers showed that the blue LEDs were more efficient in opening and closing stomata. Further, the authors also showed an increase in the number of stomata in plants grown under blue light^56^. The current study found enrichment of genes that are involved in ‘regulation of stomatal movement’ and they are upregulated in lettuce plants grown under blue light. In the current study, around 9 genes that were differentially expressed by blue light treatment were involved in flower development, which is in agreement with the results from a study by Ye et al., that revealed that blue light exposure regulates flowering^54^. Different light intensities are known to affect the plant defense-related mechanisms^24^. In the current study, blue light treatment affected many biological processes associated with the defense mechanism of plants, such as response to wounding, pathogenic bacteria, salicylic acid and hypersensitive response. There are reports of the modulation of circadian rhythm by light and dark conditions^57^, especially blue light^58^. In the current study, differentially regulated genes by blue light were enriched for ‘circadian rhythm’ related processes when compared to white and red light treatment.

The current study used RNA sequencing to explore the effect of blue, red, and white light on leaf lettuce. The assembled transcriptome was of good quality, as evident from the alignment of the RNA-Seq reads. Further, more than 50% of the assembled unigenes matched with the sequences available in well-known public databases. The differentially expressed unigenes between blue/red light and white light treatment were found to be enriched in various important physiological processes, such as photosynthesis, plant defense response, and circadian rhythm. The obtained genes can be further used to unravel their underlying roles in a specific wavelength of light during the developmental stages of lettuce.

## Supplementary Information

Supplementary Information 1.

Supplementary Information 2.

Supplementary Information 3.

Supplementary Information 4.

Supplementary Information 5.

Supplementary Information 6.

## Data Availability

The datasets generated and/or analyzed are available in the public repository under the project identifier, PRJNA739171 (https://www.ncbi.nlm.nih.gov/bioproject/?term=PRJNA739171) and the study identifier, SRP324657 (https://www.ncbi.nlm.nih.gov/sra/?term=SRP324657). The SRA accession numbers for individual experiments are as follows: SRX11181263, SRX11181262, SRX11181261, SRX11181260, SRX11181259, SRX11181258.
