# Supplementary material for: De-novo transcriptome assembly and analysis of lettuce plants grown under red, blue or white light
Source: Sci Rep. 2022 Dec 28;12:22477. doi: 10.1038/s41598-022-26344-2 (PMC9797559; doi:10.1038/s41598-022-26344-2)
Supplement: Supplementary file 1 — Supplementary Information 1. [file 41598_2022_26344_MOESM1_ESM.docx]

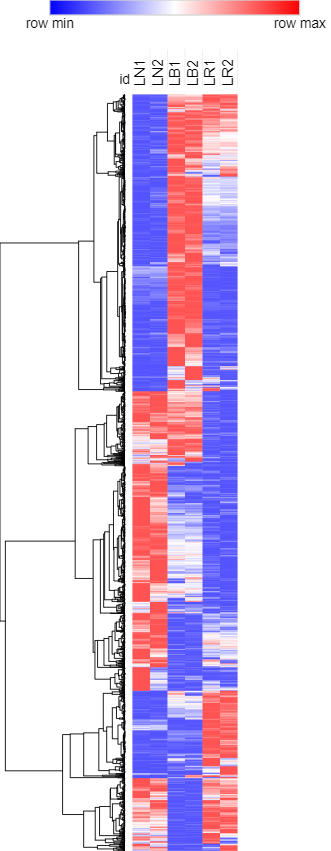


**Supplementary Figure 1: Heatmap representing the expression pattern of transcripts differentially expressed under red (LR) or blue (LB) light compared to white light (LN) treatment.** Red color indicates higher expression, whereas blue color indicates lower expression of the transcripts. The heatmap was created using Morpheus tool (https://software.broadinstitute.org/morpheus/).
